# Serological evidence of equine arteritis virus infection and phylogenetic analysis of viral isolates in semen of stallions from Serbia

**DOI:** 10.1186/s12917-017-1226-x

**Published:** 2017-11-07

**Authors:** Sava Lazić, Diana Lupulović, Delphine Gaudaire, Tamas Petrovic, Gospava Lazić, Aymeric Hans

**Affiliations:** 1 Scientific Veterinary Institute “Novi Sad”, Novi Sad, Serbia; 2 ANSES, Laboratory for Equine Diseases, Virology Unit, Dozulé, France

**Keywords:** Horse, Equine arteritis virus, Epidemiology, Phylogeny, Serbia

## Abstract

**Background:**

Equine arteritis virus (EAV) is responsible for infections in equids. It can spread easily within the horse population and has a major impact on the horse breeding industry. No EAV outbreak has ever been reported in Serbia. To determine whether EAV is nonetheless circulating there, especially in the Vojvodina region, 340 horse serum samples were subjected to serology testing to detect EAV antibodies. In parallel, semen samples from three seropositive stallions were collected to evaluate their EAV status, using RT-qPCR and virus isolation on cell culture.

**Results:**

Horse sera with EAV antibodies represented 15.88% (54/340) of the tested samples, 83.23% (283/340) being negative, and just three samples (0.89%) being uninterpretable due to cytotoxicity. Only 7.2% (10/138) of horses kept by private owners on their own property were seropositive for EAV, whereas 21.8% (44/202) of horses kept on stud farms had EAV antibodies. Phylogenetic analysis showed that the Serbian EAV isolate was most closely related to isolates from the neighbouring Hungary.

**Conclusions:**

EAV is circulating in the Serbian horse population, especially among the breeding population certainly due to the use of EAV shedder stallions since there is no surveillance programme in Serbia and only limited checks on racehorses. Moreover, phylogenetic analysis indicates that the EAV isolated from a Lipizzaner stallion in Serbia is closely related to isolates from Hungary, and together form a new cluster.

## Background

Equine arteritis virus (EAV), a member of the *Arteriviridae* family in the *Nidovirales* order [1], is the cause of an infectious disease affecting equids, including horses [2–4]. Phylogenetic analysis based on an ORF5 nucleotide sequence has grouped EAV strains into two clades: the North American group and the European group, itself divided into two subgroups named European subgroup-1 (EU-1) and European subgroup-2 (EU-2) [2]. EAV can be transmitted by respiratory or venereal routes. Although the majority of infections are subclinical, some infected animals can develop typical signs of the disease, i.e. fever up to 41 °C, depression, anorexia, oedema, nasal discharge and conjunctivitis. Young foals may lose weight and condition due to fulminating pneumonia with a lethal outcome [4]. The infection of a pregnant mare may lead to abortion, stillbirth or the delivery of a weak foal. Following primary infection, up to 70% of stallions may become persistently infected [4, 5]. Stallions shedding the virus in their semen are the reservoir of EAV and thus a key component of EAV epidemiology and transmission, since they can disseminate the virus among the horse population during breeding seasons [6].

Investigations conducted worldwide have revealed that EAV infection is present in North and South America, Europe, Australia, Africa and Asia [7–12]. Interestingly, a study performed in New Zealand concluded that there was no EVA in the New Zealand horse population [13]. The history of EVA in the Balkan region of Europe is unknown. Indeed, only one study has reported the characterisation of an EAV isolate from the semen of a Lipizzaner stallion imported in 1981 from former Yugoslavia [14].

The horse population in Serbia is estimated at around 16,000. Ten thousand horses are registered and live in Central Serbia and 6000 in Vojvodina Province, in the northern part of the country (“*Statistical Yearbook of the Republic of Serbia 2014”*, issued by the Statistical Office of the Republic of Serbia, abbreviation *STAT.YEARB.SERB. 2014*). The horses are intended for racing, working, recreational riding and carriage rides, but there is also a trade in horsemeat. Currently, neither stallions nor their sperm are monitored, and there is no active plan to prevent EVA spreading among stallions and indeed the whole Serbian horse population. There is neither an EAV vaccination policy nor any registered vaccines in Serbia. The objective of our study was thus to investigate the circulation of EAV infection and complete the molecular characterisation of EAV isolated from the horse population registered in Serbia. To our knowledge, this is the first report of the genetic characterisation of an EAV isolate from a shedder stallion bred in Serbia.

## Methods

### Blood samples

Blood samples from 340 unvaccinated horses, including 168 mares and 172 stallions, were collected for serological examination from October 2013 to October 2014. Of these, 202 horses (94 mares and 108 stallions) were from nine stud farms and four riding schools, while 138 horses (74 mares and 64 stallions) were kept on the private property of their owners. The number of horses on any one stud farm or in any one riding school ranged from 20 to 100 and from 10 to 25 respectively. Private owners kept up to seven horses on their property. After collection, the samples were kept at −20 °C until testing.

The study encompassed different types and breeds: sport horses (English thoroughbreds or part-bred horses), Arabians, Lipizzaners, Standardbred horses, French Trotters, East Bulgarian sport horses, Hungarian sport horses, Holsteiners, Hanoverians, Koninklijk Warmbloed Paard Nederland (KWPNs), Haflingers, working horses (the Nonius, Gidran, Belgian or Bosnian Mountain horse), ponies for recreational riding and grade horses used for work or raised for the meat trade. The horses ranged in age from 1 to 26 years. None of the horses from which blood was collected showed any clinical signs of the disease at the time of sampling.

### Serological examination by virus neutralisation test (VNT)

All sera samples were tested for the presence of anti-EAV antibodies by the virus neutralisation test (VNT) in accordance with chapter 2.5.10 of the OIE *Manual of Diagnostic Tests and Vaccines for Terrestrial Animals* (OIE, 2015). Briefly, serial two-fold dilutions of heat-inactivated sera from 1:2 to 1:256 were made in 96-well flat-bottom plates using Minimum Essential Media (MEM). Subsequently, an equal volume of the working dilution of stock virus with 30 to 300 tissue culture infective doses (TCID_50_) of EAV Bucyrus strain (ATCC VR - 796) was added to the plates and incubated for 1 h at 37 °C. After incubation, 100 μl of cell suspension containing about 200,000 cells/ml of RK-13 (ATCC CCL - 37) in MEM with 10% foetal calf serum was added to each well. Duplicate positive and negative serum controls were included in the assay, as were controls for serum cytotoxicity, cell viability and viral infectivity. After 72 h of incubation at 37 °C in a humid atmosphere with 5% CO_2_, the cytopathic effect (CPE) was checked under a microscope. Sera with a titre of 1:4 or higher were considered positive. Cytotoxic sera were retested using a one-day-old monolayer of RK-13 cells.

### Semen samples

During this study, 17 semen samples from three Lipizzaner stallions bred in Vojvodina Province were collected using an artificial vagina as described by Lazic et al. [15]. Four of the 11 PCR-positive semen samples were subjected to sequencing.

### Viral RNA extraction and polymerase chain reaction

RNA was extracted from the semen as described previously [16]. The eluted RNA was analysed by RT-qPCR targeting a portion of the EAV nucleoprotein (N) gene as described by Balasuriya et al. [17]. RNA from positive samples was reverse transcribed into cDNA using a SuperScript III kit (Invitrogen Corporation, Carlsbad, California, USA). The ORF5 (nucleotides 11,085–11,944 from GenBank accession number DQ846750) PCR amplification from 2.5 μl of cDNA products was performed using the PCR extender system—a kit including a high fidelity enzyme with proofreading activity (5Prime GmbH, Hamburg, Germany)—with primers 5′-TACCGCTTGGTTTTGTGGCTAT-3′ and 5′-TCACCTAAAATCCCGTCACC-3′. During the first cycle, samples were incubated at 94 °C for 2 min followed by 35 cycles under the following conditions: 94 °C for 20 s, 55 °C for 20 s, 72 °C for 2 min. The program ended with one cycle at 72 °C for 10 min.

### Sequencing and phylogenetic analyses

A total of four ORF5 PCR amplified products were sequenced (Beckman Coulter Genomics) using Applied Biosystems BigDye version 3.1. The sequences were assembled using software Ridom TraceEditPro 1.2.2., then compared to other EAV sequences using a BLAST web-based program (http://www.ncbi.nlm.nih.gov/BLAST). Nucleotide sequences were aligned in keeping with the Clustal W method. The phylogenetic trees were constructed using the Maximum Likelihood method and the tree’s statistical robustness was assessed by bootstrap resampling (1000 datasets) of the multiple alignments. Phylogenetic reconstruction was carried out using MEGA software version 5.1. [18]. The sequence described in the article was submitted to GenBank and registered under accession number KX 645659.

### Virus isolation

EAV was isolated from semen samples using an RK-13 cell line in accordance with chapter 2.5.10 of the OIE *Manual of Diagnostic Tests and Vaccines for Terrestrial Animals* (OIE, 2015). Briefly, serial ten-fold dilutions (10^−1^–10^−3^) from 1 ml of the seminal plasma were prepared in MEM, and 1 ml of each dilution was inoculated into each of two 25 cm^2^ flasks containing confluent monolayers of 3-day-old RK-13 cells. Flasks were incubated at 37 °C for 1 h then 9 ml of medium containing 0.75% carboxymethyl cellulose (CMC) was added. Flasks were checked for the appearance of any CPE on day 4 post inoculation using a 1% crystal violet solution to stain the cell monolayer. When no CPEs were detectable, a second passage was performed using new RK-13 monolayers with 1 ml of supernatant from the first passage as the inoculum and incubated for an additional 4 days.

### Statistical analysis

Statistical analyses were performed with Statistica 12 (StatSoft Inc., Dell). Differences in seropositivity between males and females; sport horses and privately-kept horses; and between breeds were calculated using the Chi-square test. A *p*-value less than 0.05 (*p* < 0.05) was considered statistically significant.

## Results

### Serological results

**Fig. 1:**
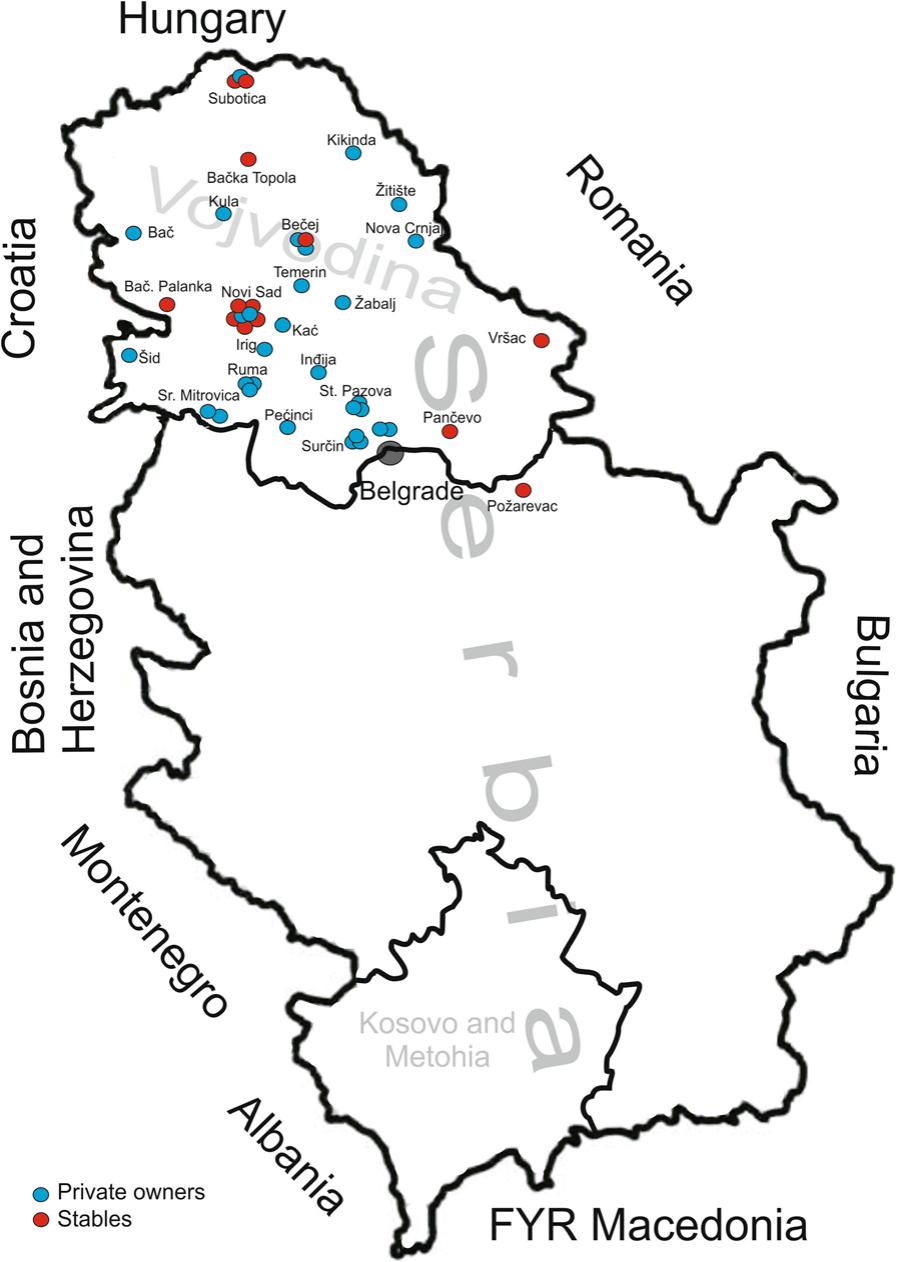
Localities of the Vojvodina region from which horse serum samples were collected

Horse serum samples were collected mainly from horses in the Vojvodina Province (northern part of Serbia), though a few were from horses in the cities of Belgrade and Požarevac in Central Serbia. The survey covered 37 locations in 21 municipalities (Fig. 1).

This study revealed that the overall percentage of EAV-seropositive horses tested was 15.88% (54/340), 83.23% (283/340) being negative. Only three samples (0.89%) were found to be cytotoxic. The antibody titres of the 54 positive sera detected ranged from 1:4 to over 1:512. The majority of animals, i.e. 70.37% (38/54) had an antibody titre value between 1:4 and 1:64, while 27.78% (15/54) reacted positively with a titre value over 1:96. Just one horse had an antibody titre higher than 1:512 (1.85%, 1/54). The population tested was composed of 168 mares and 172 stallions. The EAV seropositivity rate reached 18.45% (31/168) in mares with antibody titres from 1:4 to ≥1:512. A smaller percentage of stallions—13.37% (23/172)—were positive for EAV with antibody titres as high as 1:384. The seropositivity rate according to age category was evaluated by first dividing the equids up into three age groups. Seropositivity was seen to steadily increase with age, rising from 3.03% for younger horses (≤ 5 years old) to 34.21% for horses above 11 years old. Horses between 6 and 11 years of age had a seropositivity rate of 15.15%. These differences between the three age groups were statistically significant (*p* < 0.05).

Serological tests performed on the 202 horses bred and kept on nine stud farms or in four riding schools showed that 21.8% (44/202) were seropositive for EAV (Table 1). However, of the 13 professional structures analysed, only nos. 7, 11 and 12 had no EAV-seropositive horses. Two of these structures were riding schools and one was a stud farm breeding sport horses. Conversely, 20 out of 32 (62.5%) horses were positive for EAV on stud farm no. 6, specialising in the Lipizzaner breed (Table 1). Of seven stallions tested in stud farm no. 6, four were seropositive.

**Table 1:** Serological results of the virus neutralisation test (VNT) for equids kept in professional structures (stud farms or riding schools)

Stud farms and riding schools	Type/Breed	No. of horses tested	Positive (%)	Negative (%)	Uninterpretable (%)
1	Sport	22	2 (9.1%)	20 (90.9%)	0 (0.0%)
2	Lipizzaner	10	1 (10.0%)	9 (90.0%)	0 (0.0%)
3	Sport	24	1 (4.2%)	22 (91.6%)	1 (4.2%)
4	Sport	10	1 (10.0%)	9 (90.0%)	0 (0.0%)
5	Sport, Nonius, Lipizzaner	24	4 (16.7%)	20 (83.3%)	0 (0.0%)
6	Lipizzaner	32	20 (62.5%)	12 (37.5%)	0 (0.0%)
7	Sport	20	0 (0.0%)	20 (100.0%)	0 (0.0%)
8	Sport, Pony	17	7 (41.2%)	9 (52.9%)	1 (5.9%)
9	Lipizzaner	11	2 (18.2%)	9 (81.8%)	0 (0.0%)
10	Sport	12	3 (25.0%)	9 (75.0%)	0 (0.0%)
11	Sport, Nonius	7	0 (0.0%)	7 (100.0%)	0 (0.0%)
12	Sport	7	0 (0.0%)	7 (100.0%)	0 (0.0%)
13	Sport	6	3 (50.0%)	3 (50.0%)	0 (0.0%)
Total	/	202	44 (21.8%)	158 (77.2%)	2 (1.0%)

The percentage of EAV-seropositive equids according to the different breeds or types studied were as follows: 66.8% of ponies, 39.7% of Lipizzaners, 12.8% of sport horses and 9.1% of Nonius horses. Differences between breeds or types were statistically different (*p* < 0.05) The same analysis performed on samples from 138 horses kept by private owners on their own property showed that only 7.2% of horses were positive for EAV according to the VNT results (Table 2). Again, this difference was statistically significant (p < 0.05). Of all the tested equids kept on their owner’s private property, sport horses had the highest percentage of seropositive samples (28.57%). There were no positive results among Nonius horses, ponies, Belgian or Posavian working horses kept on their owner’s property (Table 2).

**Table 2:** Serological results of the virus neutralisation test (VNT) for equids kept by private owners on their own property

Private property	No. of private owners	Type/Breed	No. of horses tested	Positive (%)	Negative (%)	Cytotoxic (%)
A	12	Mixed breed	59	2 (3.39%)	57 (96.61%)	0 (0.0%)
B	33	Lipizzaner	54	4 (7.41%)	49 (90.74%)	1 (1.85%)
C	14	Sport	14	4 (28.57%)	10 (71.43%)	0 (0.0%)
D	3	Nonius	4	0 (0.00%)	4 (100.0%)	0 (0.0%)
E	3	Pony	3	0 (0.0%)	3 (100.0%)	0 (0.0%)
F	1	Posavian horse	3	0 (0.0%)	3 (100.0%)	0 (0.0%)
G	1	Belgian horse	1	0 (0.0%)	1 (100.0%)	0 (0.0%)
Total	67	/	138	10 (7.2%)	127 (92.0%)	1 (0.8%)

**Fig. 2:**
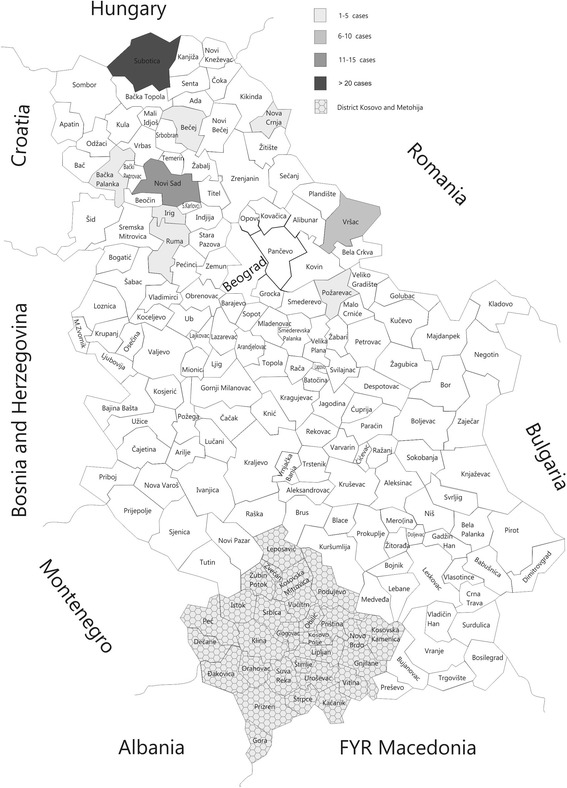
Serological results of the virus neutralisation test for EAV in horses living in Serbian municipalities

The highest number of seropositive horses was reported in Subotica, a municipality near the Hungarian border, followed by Novi Sad in the central part of the Vojvodina region and Vršac municipality near the Romanian border. The lowest number of seropositive horses was detected in the municipalities of Požarevac, Irig, Bačka Palanka, Ruma, Bečej and Nova Crnja (Fig. 2).

### Virus detection and genotyping

**Fig. 3:**
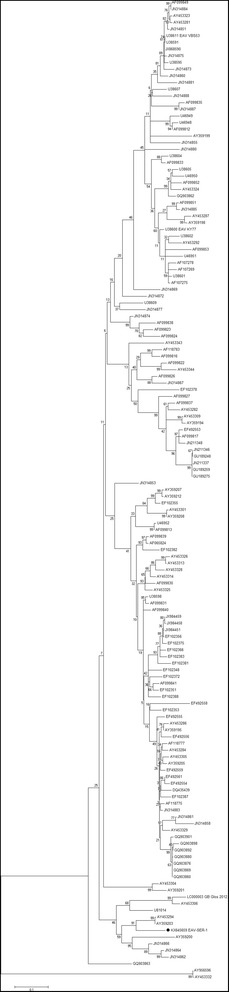
Phylogenetic analysis of the ORF5 sequences (518 nucleotides) encoded by EAV. The Serbian isolate, KX 645659 EAV-SER1, clusters with EAV strains isolated in Hungary. A Maximum Likelihood tree was created using MEGA 5 [18]. The analysis involved 147 nucleotide sequences retrieved from GenBank. The percentage of trees in which the associated taxa clustered together is shown next to the branches

Seventeen semen samples in all from three seropositive stallions were collected between 2013 and 2014. Eleven of these samples were positive for EAV by RT-qPCR. Two out of the three stallions were shedding EAV in their semen. Unfortunately, viruses could not be isolated and amplified on RK-13 cells, probably due to the delay between the collection date and the freezing date. An attempt was therefore made at sequencing viral RNA directly extracted from the semen of shedder stallions. The virus was genotyped by sequencing a 518-nucleotide portion of ORF5 encoding the virus’s GP 5. Phylogenetic analysis on this ORF5 sequence and the 145 EAV sequences retrieved from GenBank showed that the Serbian isolate, KX 645659 EAV-SER1, clusters with EAV strains isolated in Hungary [19] (Fig. 3).

## Discussion

The goals of this study were to evaluate the presence of EAV in the Serbian horse population, especially in the northern part (Vojvodina Province) as well as in Belgrade and Požarevac cities (Central Serbia), and to genotype virus from unvaccinated stallions. Indeed, no EAV vaccines are either registered or used by practitioners in Serbia. Although some evidence of EAV circulation in the Serbian horse population has been published in the past few years [15], no viral description has ever been published to date.

Our study revealed that 15.88% (54/340) of the unvaccinated Serbian horses tested had EAV antibodies. This percentage of seropositive animals is in accordance with reports from other European countries where the percentage of positive horses ranged from 11.3% to 20% [20–25]. Moreover, our data indicate that mares were more likely to be positive for EAV than were stallions (18.45% vs 13.37%), although the difference was not statistically significant.

In our survey, the seropositivity rate increased with the age of tested equids, reaching 34.21% in the older population (>11 years old). These results confirm that EAV has been circulating in the Serbian horse population for several years and are in accordance with the investigations conducted in France, Poland, Spain, Algeria, Slovenia and Bulgaria that have also described a higher seropositivity rate in older horses [11, 20, 25–27]. This may be related to the re-infection of horses during their lifetime [2]. Young horses not used for breeding may be infected by the respiratory route after contact with diseased horses within the same stable [11, 14].

The percentage of seropositive horses found in professional structures such as stud farms or riding schools, varies from 0% to 62.5%. This result can be explained by various management factors and uncontrolled mating. Indeed, other studies have reported that management efficacy and control over reproduction have a crucial impact on viral transmission [25, 27]. Interestingly, a mean of 21.9% of horses kept on stud farms were seropositive for EAV compared to 7.2% of horses kept by private owners on their own property. These results indicate that EAV appears to be less prevalent in that part of the Serbian horse population not used for breeding purposes. EAV infections in the latter population thus appear most likely to occur via the respiratory route following direct contact with infected horses. In Serbia, horses kept on stud farms are used for in-hand breeding only, since artificial insemination has not yet been implemented in the country. Moreover, the semen of stallions used for reproduction in Serbia is not tested for any sexually transmitted disease such as EAV. Stallions shedding EAV in their semen may therefore transmit the virus to mares during mating and those covered mares are likely to transmit the virus to other horses in the stable by the respiratory route following direct contact. Our data indicate that the risk to the breeding population of contracting EAV is higher than the risk to horses kept by their owners on private property, reinforcing the importance of good practice as well as a strict application of health measures when managing horse breeding on stud farms.

Interestingly, the EAV isolate obtained from a Lipizzaner stallion appeared to differ from those available in GenBank. However, this Serbian isolate—KX 645659 EAV-SER1is closely related to a virus isolated in the neighbouring Hungary [19]. Moreover, our phylogenetic analysis showed that the latest strain described by Steinbach et al. [28] is also closely related to both a Hungarian strain [29] and a strain isolated in Kentucky, USA [30]. Taken together, the EAV strains described recently clustered together to form a new distinct group of viruses originating in the central European region.

## Conclusions

This study is the first equine serological survey conducted in Serbia that encompasses data on type/breed, age, titre level and EAV geographical distribution. EAV is shown to be currently circulating in the Serbian horse population since 15.88% (54/340 tested animals) of the animals tested had EAV antibodies despite the fact that there are no registered vaccines and no vaccination policy in Serbia. Moreover, the sequence analyses and phylogenetic characterisation of EAV from a persistently infected stallion is also reported for the first time in Serbia. The isolate characterised here is closely related to the strain isolated in Hungary and, to a lesser extent, to the isolate described in 2015 by Steinbach et al. in the United Kingdom [28]. Moreover, these strains group together to form a new cluster.

Serbian authorities have not yet initiated a programme to control the spread of EAV within the horse population. Serological testing is basically carried out once a year in the case of racehorses; before an agricultural fair in the case of working horses; or on the owner’s personal request, usually prior to mating. Clinical cases are neither registered nor declared to the competent authorities. There is furthermore a need to improve the cooperation of horse owners and veterinarians with official veterinary authorities in order to improve the reporting of actual or suspected cases of EAV and to clarify the epidemiology of the infection.

Our data show that horses used for breeding in Serbia are more likely to test positive for EAV than the rest of the horse population, probably due to the use of EAV shedder stallions. To prevent the further spread of EAV in the Serbian breeding horse population, it is essential to implement effective strategies and a surveillance programme to check the EAV carrier status of stallions.

## Abbreviations

EAV: Equine arteritis virus
EU-1: European subgroup 1
EU-2: European subgroup 2
EVA: Equine Viral Arteritis
RT-qPCR: Reverse transcriptase quantitative polymerase chain reaction
VNT: Virus neutralisation test
